# Diagnostic Accuracy Study Comparing Hysterosalpingo-Foam Sonography and Hysterosalpingography for Fallopian Tube Patency Assessment

**DOI:** 10.3390/jcm10184169

**Published:** 2021-09-15

**Authors:** Julia Ramos, Cinzia Caligara, Esther Santamaría-López, Cristina González-Ravina, Nicolás Prados, Francisco Carranza, Víctor Blasco, Manuel Fernández-Sánchez

**Affiliations:** 1 IVIRMA Sevilla, Avenida Américo Vespucio 19, ES-41092 Seville, Spain; julia.ramos@ivirma.com (J.R.); cinzia.caligara@ivirma.com (C.C.); esther.santamaria@ivirma.com (E.S.-L.); nicolas.prados@ivirma.com (N.P.); francisco.carranza@ivirma.com (F.C.); victor.blasco@ivirma.com (V.B.); manuel.fernandez@ivirma.com (M.F.-S.); 2 Fundación IVI, Instituto de Investigación Sanitaria La Fe, ES-46026 Valencia, Spain; 3 Department of Molecular Biology and Biochemical Engineering, Universidad Pablo de Olavide, ES-41013 Seville, Spain; 4 Department of Surgery, Universidad de Sevilla, ES-41004 Seville, Spain

**Keywords:** tubal patency, hysterosalpingo-contrast sonography, hysterosalpingo-foam sonography, hysterosalpingography, HyFoSy, HSG

## Abstract

Introduction: Simplified ultrasound-based infertility protocols that appear to provide enough information to plan effective management have been described. Thus, the objective of this study is to compare the diagnostic accuracy of the hysterosalpingo-foam sonography (HyFoSy) in tubal patency testing with the traditional hysterosalpngography (HSG) for establishing a new diagnostic strategy in infertility. Material and Methods: Prospective observational diagnostic accuracy was performed in a private fertility clinic in which 106 women undergoing a preconceptionally visit were recruited. All of them had low risk for tubal disease, had performed an HSG and were negative for *Chlamydia trachomatis* antibody. Main outcome measures were tubal patency and pain grade. Results: Evaluation of tubal patency by HyFoSy showed a total concordance with the results of the previous HSG in 72.6% (*n* = 77), and a total discordance for 4.7% (*n* = 6), with the inter-test agreement Kappa equal to 0.57, which means moderate concordance. Among the patients, 59.1% did not report pain during the procedure, while the remaining 48.1% indicated pain in different degrees; patients usually report less pain and only 6.6% described more pain with HyFoSy than with HSG (OR 6.57 (CI 95% 3.11–13.89)). Clinical outcomes after performing HyFoSy were not affected. Conclusions: HyFoSy is in concordance with HSG regarding tubal patency results and it is a less painful technique than HSG. HyFoSy is more economical and can be performed in an exam room only equipped with an ultrasound scanner. Based on these results, HyFoSy could be the first-choice diagnostic option to assess tubal patency in patients with low risk of tubal disease.

## 1. Introduction

Imaging diagnostics are an essential part of contemporary medicine. Ultrasound-based examination plays a special role in gynecology and its use has been increasing, particularly in the evaluation of infertility. Traditionally, the cornerstones of investigation for an infertile couple have been grouped into several testing categories, with the evaluation of uterine architecture and tubal patency being one of the most important ones [1]. Tubal abnormalities are seen in 25–35% of female subfertility patients [1]. Tubal disease encompasses a range of pathologies, with pelvic inflammatory disease (PID) as the most common cause; it is usually asymptomatic, and its main causal agent is *Chlamydia trachomatis* [2]. Other possible causes of tubal infertility are endometriosis, previous pelvic surgery, fibroids and pelvic tuberculosis [3].

Laparoscopy with chromopertubation (LC) is the gold-standard technique for tubal assessment, but because it is an invasive, expensive and riskier technique [1,4] it was replaced by HSG, which has been used for decades. This latter procedure has lower diagnostic accuracy than LC [5], is unable to detect abnormal ovaries or myometrium and involves radiation exposure, discomfort or even abdominal pain [6]. Considering the disadvantages of HSG, hysterosalpingo-contrast sonography (HyCoSy) was introduced as an alternative for outpatient tubal assessment as it avoids the previously mentioned risks. HyCoSy, using a mixture of air and saline solution, has an accuracy comparable to that of HSG [7], allows the real-time observation of the pelvic organs through ultrasound and it performs better than HSG when detecting abnormalities in the uterine cavity [8]. However, HyCoSy is more operator-dependent than the two other techniques. At this point, it is important to highlight that all these methods have technical limitations, so scientific societies recommend further evaluation with a second technique in case of abnormal results to establish a correct diagnosis and find the best treatment strategy for the patient [1].

Different contrast media have been proven effective but have become no longer available for different reasons [9]. In 2007, a non-embryotoxic gel known as ExEm^®^ gel (GynaecologIQ, Delft, The Netherlands) was developed. Because ultrasound scans (US) of good-quality at an acceptable price were obtained, hysterosalpingo-foam sonography (HyFoSy) has been established as a safe and less painful alternative and has become widely adopted in infertility clinics and outpatient settings, shortening waiting times for treatment initiation [10]; moreover, its feasibility, tolerability and safety have been already demonstrated [11,12,13].

The ability to perform most infertility diagnoses by ultrasound in the ambulatory setting is not only attractive and beneficial to patients, but also to the healthcare system. Thus, the objective of this study is to estimate the diagnostic accuracy of HyFoSy in tubal patency testing and compare it to the accuracy of traditional HSG for establishing a new diagnostic strategy in infertility treatment.

## 2. Materials and Methods

### 2.1. Patients

**Figure 1:**
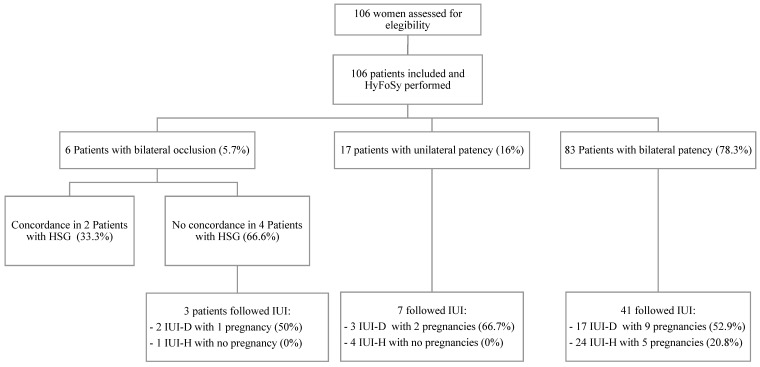
Flowchart of eligible participants. HyFoSy: hysterosalpingo-foam sonography; HSG: hysterosalpingography; IUI: intrauterine insemination; IUI-D: intrauterine insemination with donor semen; IUI-H: intrauterine insemination with husband or partner’s semen.

This study was approved by the institutional Ethics Committee of the Hospital Universitario Virgen Macarena (Seville, Spain) and all patients signed an informed consent form (internal Ethics Committee number 2071). One hundred and six patients were recruited between June 2013 and February 2017 in our fertility center (Figure 1). The inclusion criteria were having a previous HSG performed in less than a year, a negative serology for IgG and IgM *Chlamydia trachomatis* and being patients with low risk for tubal disease. Risk for tubal disease was assessed performing a US and exhaustive anamnesis (no evidence of PID or other pelvic diseases such as hydrosalpinx, adnexal pathology, previous abdominal surgery or endometriosis).

### 2.2. Technique

The HyFoSy procedure was always performed by the same trained clinicians and under the same conditions. Prior to the examination, a 2-dimensional ultrasound (IUI-US) was achieved to assess pelvic anatomy. All US were performed using a General Electric Voluson 730 proV system equipped with a volumetric 4–9 MHz endovaginal probe (IC5-9H H40422LL).

The foam contrast was prepared and performed at room temperature as previously described [14]. In most cases, we used the GIS catheter (Smith Medical, United Kingdom), which is included in the ExEm^®^ Foam kit, but in patients in whom it was not possible to introduce it, we needed an alternative one, such as the Gynetics^®^ (#4219 Emtrac Delphin Embryo transfer catheter) or Kitazato^®^ (4.7Fr 230 mm Trial EC-PRO Catheter), which are softer and narrower. The slow introduction of 5 mL of foam in one bolus into the endometrial cavity was enough to achieve correct visualization. We performed a longitudinal uterus ultrasound analysis to evaluate that foam was passing through it and not into the vagina. Then, we studied a transverse section of the uterus to locate the tubes. Finally, we checked for foam dispersion around the ovaries and inside the peritoneal cavity. The gel usually maintained its echogenicity long enough, between 5 and 12 min, to allow image acquisition. We defined tubal patency as gel foam spillage from the fimbria ending, seen as fluid flow surrounding the ovary and its collection in pelvis. Distal tubal blockage was defined as absence of spillage. In the event of apparent cornual blockade, we proceeded following the suggestion that adding an additional foam injection may correct the tubal spasms [15].

At the end of the process, patients had to fill out a questionnaire about the experienced pain. Patients had to choose a value from 0 to 10 using visual analogue scale (VAS) to describe the pain experienced in both techniques: HyFoSy and HSG. No analgesics were indicated prior to the performance of the technique.

### 2.3. Clinical Outcomes

After tubal patency diagnosis, fifty-one patients underwent intrauterine inseminations (IUI) as shown in Figure 1. A maximum of 4 IUI were performed before being referred to an in vitro fertilization (IVF) treatment. Clinical pregnancy rate calculated as the percentage of pregnancy women per intrauterine insemination with donor semen (IUI-D) and intrauterine insemination with husband or partner’s semen (IUI-H) performed were compared with our indicators to validate the tubal patency diagnosis.

### 2.4. Statistical Analysis

Statistical analysis was performed according to tubal patency and pain grade. Tubal patency was described as bilateral patency, unilateral patency, or no patency. Pain results were assessed using the visual analogue scale (VAS) and classified into four groups to be analyzed as an ordinal categorical outcome according to Engels et al. [13]: no pain (VAS 0), mild pain (VAS 1–3), moderate pain (VAS 4–6) or severe pain (VAS 7–10). The side of the patency was noted to distinguish agreement between techniques. For descriptive statistics, data are presented as the means and the 95% IC of their difference. Each comparison generated a different *p*-value. In all cases, *p*-values ≤ 0.05 were considered significant. Wilcoxon matched-pairs signed-ranks test was used to analyze the data. There is no published data about correlation between HyFoSy and HSG results or patient outcome that can help us calculate beforehand the sample size. Because it is an exploratory study, we included a minimum of 100 patients. We analyzed concordance with the weighted Kappa index [16]. We used the Statistical Package for Social Sciences version 22 (SPSS, USA) software for data analysis.

## 3. Results

The characteristics of the patients (female age, BMI and infertility duration) are summarized in Table 1.

The evaluation of tubal patency by HyFoSy showed a total concordance with the results of the previous HSG in 72.6% (*n* = 77) of the cases (Table 2). On the other hand, we also described a total discordance for 4.7% of the procedures (*n* = 5); 3.8% (*n* = 4) were diagnosed with a bilateral obstruction by HyFoSy while both of their tubes were patent according to HSG test, and 0.9% (*n* = 1) was diagnosed with unilateral patency, but in a different tube depending on the test performed. The inter-test agreement Kappa was 0.57, showing a moderate concordance.

Concerning the catheter used during the technique, 57.5% of the procedures were performed with the cannula corresponding to the ExEm^®^ Foam kit, 37.5% with a Kitazato^®^ and 0.05% with a cannula belonging to Gynetics^®^.

During the period of the study, 59.1% of the patients did not report pain during the procedure, while the remaining 48.1% indicated pain in different degrees, as shown in Table 3. When patients were asked about the pain, they had experienced during previous HSG, 50% of the responses indicated the same pain, 43.4% answered that they suffered lees and only the remaining 6.6% described more pain with HyFoSy than with HSG (OR 6.57 (CI 95% 3.11–13.89)).

An important point to be analyzed was the number of adverse events and it is noteworthy that no infections were reported in any patient after the HyFoSy procedure. Four vasovagal reactions were reported, in one case without pain and in three cases with mild pain during the process. A painless spotting episode was also described.

Finally, clinical results are summarized in Table 4. Compared with our general population that underwent an IUI-D during the study period, clinical pregnancy rates were better after HyFoSy (27.3% vs. 20.8%, *p* = 0.453); for IUI-H, the same trend is observed (10.3% vs. 14.8%, *p* = 0.496), although, as expected, outcomes were lower than with IUI-D.

## 4. Discussion

An optimal initial infertility screening protocol would be a process that is diagnostically accurate, profitable, reliable and as least invasive as possible. In addition, the investigation should provide the physician with useful prognostic information regarding possible future treatment. Currently, the extensive use of invasive procedures such laparoscopy or hysteroscopy are the standards at many fertility centers but advances in gynecological ultrasonography have shown that ultrasounds could replace routine invasive procedures. An ultrasound-based approach would make the basic infertility diagnosis less time-consuming and less expensive, but at the same time more acceptable to most patients; thus, it is vital for gynecologists to implement modern non-invasive ultrasound modalities in daily practice.

As we stated previously, tubal disease is an important cause of infertility and tubal patency testing is an important part of the infertility investigation [1]. The disadvantages associated with conventional tests such as LC and HSG paved the way for the development of HyFoSy which is described in comparison with the other techniques as just as accurate, more patient-friendly and cost-effective and less painful; in addition, it uses a non-embryotoxic contrast and does not require to refer patients to another center for tubal patency evaluation as it could normally be performed at the fertility center itself, reducing the waiting times for starting an assisted reproductive treatment. Finally, indications for this technique include a basic infertility study, when intrauterine insemination or programmed intercourse are prescribed as preferent treatments and to verify that the tubes are occluded after a surgical procedure [17].

Bearing in mind these considerations, we designed a diagnostic accuracy study comparing a previous HSG with the results of the current HyFoSy test. Previous results have shown a total concordance with LC [18] and a higher proportion of tubes were classified as patent when compared with HyCoSy [12]. In addition, HyFoSy provided a more accurate diagnosis compared to HyCoSy with saline solution when both techniques were confronted with LC [19]. As far as we know, this is the first study whose results show a high enough degree of concordance between the two techniques analyzed to be able to introduce HyFoSy as a first-line clinic tubal patency test [20]. This statement is confirmed in cases where additional testing is needed if the results are abnormal during the first evaluation. In this sense, we would have needed to perform a second complementary test in those patients who presented bilateral tubal obstruction with HyFoSy, but as these women already had a previous HSG, we could confirm the initial diagnosis in two of the cases without performing a third procedure. In our case, the percentage of inconclusive results after HyFoSy is lower than in other studies [11,14]; the reason may be found in the implementation of a proper learning curve, which will facilitate the establishment of this diagnostic strategy by reducing the cases that need to be confirmed.

In attempts to overcome the inability to examine the whole course of the fallopian tube in one scanning plane [21], other techniques such as 3-dimensional ultrasound (3D-US) and doppler have been assessed instead of 2D-US to improve visualization while performing HyFoSy; however, up to date, it is therefore questionable whether these techniques are of additional value for HyFoSy. It has been reported that, with or without doppler, 3D-HyFoSy does not seem to offer benefits above real-time 2D-HyFoSy performed by an experienced ultrasonographer, so we could assume that HyFoSy appears to be an accurate and well-tolerated first-line diagnostic procedure and 3D-HyFoSy technique is helpful for a less qualified operator [19,22]. Therefore, the fact of having used 2D-HyFoSy, along with the fact that the procedure has been performed by a qualified sonographer, could also explain why fewer confirmatory tests are needed in our study than in other of a similar nature. Just as important as the visualization technique, is the choice of the appropriate cannula to perform the procedure; some authors have recommended the use of a pediatric Foley balloon catheter to prevent reflux [15] but based on our experience, the use of an intrauterine balloon-less catheter is more useful, while it is visible in the uterine cavity, in case of having difficulties with the ExEm^®^ Foam catheter as they are less painful and cheaper.

Once the precision of HyFoSy was accepted for the assessment of tubal patency, we focused on patient’s pain. Our results showed that, in general, HyFoSy is a less painful technique compared with conventional HSG; these data are consistent with those of a previous study in that the average time required to perform the procedure is related to the intensity of pain [11]. However, and despite our results being encouraging regarding the pain associated to HyFoSy, they are not as good from the point of view of adverse events, since we reported a higher incidence of vasovagal reactions compared to other studies [13]. It is important to remember that these patients recover quickly after changing position.

Lastly, we confirmed that the clinical pregnancy rates from intrauterine inseminations carried out after performing HyFoSy were not affected by an incorrect diagnosis since our outcomes were like those observed in daily clinical practice, when HyFoSy was previously used to diagnose tubal patency. In addition, the studies published to date have reported a spontaneous pregnancy rate that ranges between 19–30% for a period of approximately 6 months [10,23].

In summary, ultrasound fertility assessment is an accurate choice for a first line infertility workup and its use has been rising. This has allowed the development of treatment strategies such as Fertiliscan©, based on the realization of a high-quality 3D-US that involves the assessment of tubal patency by performing HyFoSy [24]. This integral approach is targeted to address specific issues concerning the fertility potential of the woman. 

## 5. Conclusions

HyFoSy is a suitable technique to assess tubal patency. Our results suggest that it could be considered a promising alternative for HSG regarding accuracy and effectiveness in patients at low risk for tubal disease. Furthermore, HyFoSy is cost-effective, less time-consuming, well-tolerated, can be performed in an exam room equipped only with an ultrasound scanner, reduces waiting times and might not affect clinical outcomes after an assisted reproductive treatment. The establishment of an appropriate diagnostic strategy that includes qualified staff and the most effective ultrasound technique to carry out the procedure, has allowed us to reduce the cases that need to be confirmed.

## Figures and Tables

**Table 1:** Patient characteristics.

	Mean (*n* = 106)	Standard Deviation
Age (y)	34.71	±3.68
Body mass index (kg/m^2^)	24.11	±4.54
Infertility duration (y)	2.30	±1.70

**Table 2:** Patency results.

*n* = 106 Patients		Patency during HyFoSy		
		Bilateral Obstruction (*n* = 6; 5.7%)	Unilateral Obstruction (*n* = 17; 16%)	Bilateral Patency (*n* = 83; 78.3%)
Patency during HSG	Bilateral obstruction (*n* = 5; 4.7%)	2 (1.9%) *	3 (2.8%)	0 (0.0%) **
	Unilateral obstruction (*n* = 15; 14.2%)	0 (0.0%)	3 (2.8%) *	11 (10.4%)
			1 (0.9%) **	
	Bilateral patency (*n* = 86; 81.1%)	4 (3.8%) **	10 (9.4%)	72 (67.9%) *

Wilcoxon matched-pairs signed-ranks test (*p* < 0.001); inter-test agreement Kappa index (0.57); * total concordance; ** total discordance.

**Table 3:** Pain results.

*n* = 106 Patients		Pain during HyFoSy			
		No Pain (*n* = 55; 51.9%)	Mild Pain (*n* = 44; 41.5%)	Moderate Pain (*n* = 6; 5.7%)	Severe Pain (*n* = 1; 0.9%)
Pain during HSG	No pain (*n* = 37; 34.9%)	31 (29.2%)	6 (5.6%)	0 (0.0%)	0 (0.0%)
	Mild pain (*n* = 22; 20.8%)	2 (2.0%)	19 (17.9%)	1 (0.9%)	0 (0.0%)
	Moderate pain (*n* = 33; 31.1%)	14 (13.2%)	17 (16.0%)	2 (2.0%)	0 (0.0%)
	Severe pain (*n* = 14; 13.2%)	8 (7.5%)	2 (2.0%)	3 (2.8%)	1 (0.9%)

Wilcoxon matched-pairs signed-ranks test (*p* < 0.001).

**Table 4:** Clinical outcomes after tubal patency diagnosis.

	IUI-D (*n* = 22)	IUI-H (*n* = 29)	Total (*n* = 51)
Clinical pregnancy rate at first IUI	27.3%	10.3%	17.7%
Clinical pregnancy rate after two IUI	45.5%	17.2%	29.4%
Cumulative clinical pregnancy rate	54.6%	17.2%	33.3%

IUI-D: intrauterine insemination with donor semen; IUI-H: intrauterine insemination with husband or partner’s semen.
